# P-1059. *In vitro* Activity of Gepotidacin against *Klebsiella pneumoniae*, Including Molecularly Characterized ESBL and Carbapenemase positive Subsets Causing Urinary Tract Infections in the United States (2019-2022)

**DOI:** 10.1093/ofid/ofae631.1248

**Published:** 2025-01-29

**Authors:** Rodrigo E Mendes, Abigail Scullin, Hank Kimbrough, Cory Hubler, Renuka Kapoor, Didem Torumkuney, S J Ryan Arends

**Affiliations:** JMI Laboratories, North Liberty, Iowa; Element Materials Technology/Jones Microbiology Institute, North Liberty, Iowa; Element Materials Technology/Jones Microbiology Institute, North Liberty, Iowa; Element Materials Technology/Jones Microbiology Institute, North Liberty, Iowa; GSK, Atlanta, Georgia; GSK, Atlanta, Georgia; JMI Laboratories / Element, North Liberty, Iowa

## Abstract

**Background:**

Gepotidacin (GEP) is a novel, bactericidal, first-in-class triazaacenaphthylene antibacterial that inhibits bacterial DNA replication by a unique mechanism of action, distinct binding site, and provides well-balanced inhibition (for most uncomplicated urinary tract infection [uUTI] uropathogens and *Neisseria gonorrhoeae*) of two different type II topoisomerase enzymes. GEP recently completed two phase 3 trials for the treatment of uUTI. This study reports the activity of GEP and other oral antibacterials against *K. pneumoniae*, including molecularly characterized ESBL and carbapenemase producing isolates collected from UTI patients in the US.

**Figure:**
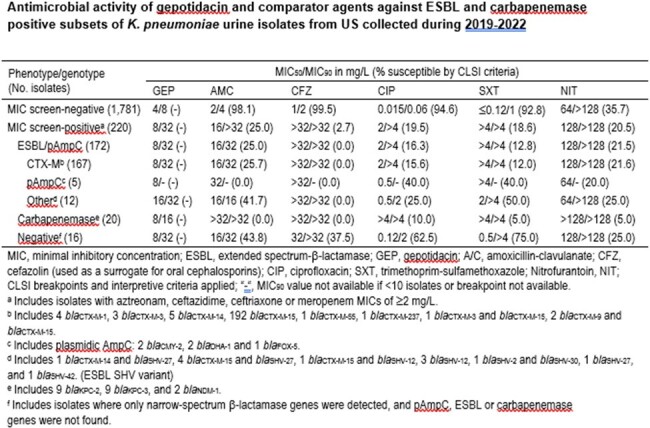

**Methods:**

A total of 2,001 *K. pneumoniae* were collected from 73 sites. CLSI methods were used for both susceptibility testing and MIC interpretation. Isolates with aztreonam, ceftazidime, ceftriaxone or meropenem MIC of ≥2 mg/L were subjected to genome sequencing, and screening of plasmid-mediated AmpC (pAmpC), ESBL, and carbapenemase genes.

**Results:**

A total of 11.0% (220/2,001) *K. pneumoniae* were screened for β-lactamase genes (Table). Majority of these isolates carried *bla*_CTX-M_ alone (78.2%; 172/220), and a small number carried combinations of *bla*_CTX-M_ and ESBL variants of *bla*_SHV_ (2.7%; 6/220), *bla*_SHV_ alone (2.7%; 6/220), or pAmpC alone (2.3%; 5/220). Among screened isolates, 9.1% (20/220) carried carbapenemases. GEP had MIC_50_ and MIC_90_ of 4 mg/L and 8 mg/L, respectively, against isolates that did not meet the MIC criteria for screening of β-lactamases. Other oral agents showed susceptibilities of 92.8–99.5% against this group, except for nitrofurantoin (35.7%). GEP had an MIC_90_ of 32 mg/L against screened strains carrying ESBL and pAMPC, and an MIC_90_ of 16 mg/L against carbapenemase positive isolates. Other oral agents had limited activity (< 42%) against strains carrying ESBL and/or pAMPC.

**Conclusion:**

GEP showed activity against UTI-causing *K. pneumoniae* in US medical centers, including against isolates carrying ESBL, pAmpC and/or carbapenemases. These data support further clinical development of GEP as a potential treatment option for uUTI caused by *K. pneumoniae* including when other oral treatment options have limited activity due to drug resistance.

**Disclosures:**

**Rodrigo E. Mendes, PhD**, GSK: Grant/Research Support **Renuka Kapoor, PhD**, GSK: Employee|GSK: Stocks/Bonds (Public Company) **Didem Torumkuney, PhD**, GSK: Employee|GSK: Stocks/Bonds (Public Company)

